# Foraging actively can be advantageous in heterogeneous environments

**DOI:** 10.1098/rsbl.2025.0153

**Published:** 2025-07-02

**Authors:** Dylan J. Padilla Perez, John M. VandenBrooks, Marla B. Sokolowski, Michael J. Angilletta Jr

**Affiliations:** ^1^ School of Life Sciences, Arizona State University, Tempe, AZ 85287, USA; ^2^ School of Applied Sciences and Arts, Arizona State University, Tempe, AZ 85287, USA; ^3^ Department of Ecology and Evolutionary Biology, University of Toronto, Toronto, Ontario, Canada

**Keywords:** colonization, dispersal, foraging gene, plasticity

## Abstract

A wealth of evidence indicates that behavioural polymorphism is widespread in nature. While some organisms search for food by moving almost continuously throughout their environment, other organisms forage in one place for long periods of time. Although such a dichotomy has been previously documented in *Drosophila melanogaster*, the question remains which foraging strategy is better suited to maximize energy intake in a particular environment. We designed an experiment to evaluate whether the configuration of food in the environment alters the foraging behaviour of two larval strains. Assuming that one of the strains acquires more food than the other in a given environment, we examined whether variation in growth occurred between them. Our results indicate that foraging behaviour is a plastic trait, shaped by the configuration of food in the environment. Regardless of the foraging strategy, we found that larvae generally increased their locomotion when food was patchy rather than clumped. Even though we observed that some individuals actively sought food while others stayed foraging at nearby sites, we found no differences in growth rate between them. However, we suggest that foraging actively may be advantageous in polymorphic populations because such behaviour facilitates local adaptation via founder effect and gene flow.

## 1. Introduction

A central goal of behavioural ecology is to determine how organisms exploit food in a given environment [1]. In nature, food is distributed in patches that vary in size and density over time. In response to such environmental heterogeneity, organisms can adopt various foraging behaviours that maximize their acquisition of energy [2]. In a behaviourally polymorphic population, some organisms move frequently while foraging, abandoning patches of food before fully depleting them. By contrast, other organisms minimize their locomotion, rarely abandoning patches of food. For example, a seemingly tireless hummingbird may actively visit flowers in search of nectar, while a kingfisher perches by stream until a fish swims into view. Rather than a true dichotomy, these extreme behaviours lie along a continuum of foraging strategies, from mobile to sedentary [3–5].

When food varies over space and time, theoretical models of the optimal foraging strategies predict that organisms should use a simple decision rule for leaving a patch of food [1,6,7]. An individual should leave a patch only when the net gain from moving exceeds the expected gain from remaining at the site. Indeed, lizards *Pedioplanis lineoocellata* increased their exploratory behaviour when the cost of foraging increased as a result of scarce vegetation cover in the environment, which translates into a low abundance of invertebrates that are part of the lizards’ diet. Alternatively, lizards of the same species foraged in nearby sites when the cost of foraging decreased as a result of both abundant vegetation and food in the environment [8]. This observation aligns with the results of a recent study on the exploratory behaviour of *Drosophila melanogaster* under conditions of food deprivation; Hughson *et al.* [9] showed that flies invested more time seeking feeding sites after prolonged food deprivation. Based on the existing evidence, one can predict which behaviour is better suited for an organism to maximize energy intake in a particular environment.

If variation in foraging behaviour affects the energetic benefits and costs, then growth rate, body size and reproductive output should vary among organisms that forage differently. This expectation is supported by the idea that different behavioural strategies determine the life histories of organisms by limiting their acquisition and allocation of energy [10–13]. An allocation trade-off suggests that using more energy for one function results in less energy remaining for other functions. Thus, an individual that acquires a surplus of energy may grow and develop faster than one with restricted energy stores [13,14]. Consistent with these predictions, a recent investigation indicates that larvae of *D. melanogaster* that forage more actively are more likely to survive and develop faster in a nutrient-poor environment [15]. However, fecundity did not differ between individuals that vary in foraging behaviour regardless of food abundance [16]. These findings highlight the need for further investigation into the expected life-history variation among organisms that forage differently.

The well-characterized behavioural polymorphism of *D. melanogaster* provides an excellent opportunity to experimentally test some predictions about the relationship between foraging behavior and life history. Previous evidence suggests that a gene of major effect influences the distance that fly larvae travel while foraging, with two allelic variants: rover ($for^{R}$) and sitter ($for^{S}$). Rovers travel significantly longer distances than sitters when exposed to a nutrient-rich substrate [17]. Accordingly, we hypothesized that the expression of larval foraging behaviour depends on the spatial configuration of food in the environment. In a patchy environment, for example, we predicted that rovers would explore a larger proportion of area and travel longer distances than sitters. This prediction is consistent with recent evidence suggesting that rovers dispersed more than sitters when the total amount of food increased with the number of patches [18]. However, this difference would become negligible in environments with a clumped configuration of resources. Generally, both rovers and sitters would cover more area and travel longer distances in a patchy environment compared to an environment with a clumped configuration of resources. Our second hypothesis was that foraging behaviour influences the energetic benefits and costs, leading to variation in the life history of the larvae. Specifically, we expected sitter larvae to grow faster than the rover larvae in an environment with a clumped configuration of food, while the opposite pattern would be the case in a patchy environment. Additionally, we predicted that growth differences would be more pronounced when food was clumped rather than patchy.

## 2. Material and methods

### (a) Fly strains

We used the rover ($for^{R}$) and sitter ($for^{S}$) wild-type strains in our experiments. These strains share X and isogenized third chromosomes from the rover strain and differ in their second chromosomes where the foraging gene is located. An isogenic chromosome refers to a situation where individuals in a population share nearly identical genetic make-up. The pair of wild-type second chromosomes in the rover lab strain originated from a population of flies collected in a compost bin near Toronto, Ontario, Canada [19]. The pair of wild-type second chromosomes in the sitter strain originated from a wild-type Oregon R strain, a standard wild-type laboratory strain. Standard genetic complementation and deletion analyses showed that the wild-type lab rover and sitter strains are allelic (same gene, *foraging* and same effect on larval behaviour) to the orchard population-derived rover and sitter strains [20,21].

We housed the strains at 25°C, in a 12 : 12 hour light/dark cycle at 60% relative humidity with lights on at 07.00. We reared the flies in ∼240 ml round-bottom plastic bottles, with a standard yeast–sugar–agar medium Anreiter *et al.* [22]. Before the beginning of each experimental trial, we transferred the flies into empty bottles and capped them with grape plates containing a small amount of dry active yeast to stimulate reproduction. After 22 h, we removed the grape plates from the bottles and discarded all larvae from each plate with a dissecting probe. We then incubated the eggs that remained in the grape plates for 4 h in standard conditions as described earlier. After 4 h in standard conditions, we picked L1 larvae of each strain from the grape plates and placed them individually in food plates (i.e. yeast–sugar–agar medium). Lastly, we collected third-instar larvae about 10 h before wandering Anreiter *et al.* [22]. The wandering behaviour consists of a period of increased locomotor activity by the larvae when searching for pupation site.

### (b) Design of environments

We estimated the locomotion of larvae in two types of environments by computing the proportion of area covered and the total distance travelled while foraging. One environment consisted of yeast paste regularly distributed in patches on a matrix of *Drosophila* agar (1%) medium, while the other one consisted of a single yeast clump placed on the same agar medium. We prepared these environments in 100 × 15 mm Petri dishes capped with standard lids. To make the patchy environment, we used a 12 ml insulin syringe to dispense small drops of dry active yeast mixed with water at a 1 : 2 ratio (weight to volume) on the agar matrix. Patches were separated from each other by a distance of 20 mm on the vertical plane and 40 mm on the horizontal plane, creating a rectangular grid pattern whose vertices consisted of 15 patches (electronic supplementary material, figure S1). We used the same method to make the environment with a clumped configuration of food, but this time we poured the paste in such a way that a food clump formed in the centre of each plate. To design both environments, we used moulds to assure that the configuration of patches was consistent (electronic supplementary material, figure S1). We also made sure that the volume of food used (2 µl) was the same among plates.

### (c) Locomotor performance assay

After setting up the test plates, we released a single L3-foraging larva into each plate capped with its lid, randomizing the combination of strain, environment and position where the larva was released. Each larva was released in the centre of four possible quadrants that were randomized before the beginning of the experiments. To randomize these factors, we used the ‘*sample*’ function available in the free software R v. 4.3.2 [23], which enabled us to pick a sample of a specified size (n=1 in this case) from a vector of predefined elements (e.g. a vector of two characters: ‘rover’ and ‘sitter’). We then transferred the plates to an incubator set up at 25°C and 60% relative humidity. After a period of 1 h, we recorded the larvae for 30 min, using a camera (Canon EOS Rebel T7 EF-S 18-55 mm IS II) held 30 cm above the plates. In each trial, we recorded four plates simultaneously as indicated in figure 2. This experiment yielded data for n=92 larvae; 46 larvae of each strain were randomly divided into two groups (n=23) to be tested in environments where the configuration of food was clumped or patchy.

To analyse the video recordings, we used the free software AnimalTA v. 2.2.1 [24]; a video-tracking software that enabled us to analyse videos recorded under the same conditions. This program identifies the target individual using an adaptive thresholding method, which makes the target animal look darker than their local environment. This way, one can produce better tracking results than the frequently used background subtraction method, especially when the background is changing. To make sure that the output of the program was reliable, we manually double-checked and corrected the tracking results of the videos.

### (d) Growth assay

To quantify growth, we let larvae develop to the L3-stage in Petri dishes capped with their lids containing the standard yeast–sugar–agar medium [22]. We measured growth as the difference between the initial mass and the final mass of individual larvae after a 24 h period under standard conditions. To record the initial mass of a larva, we gently washed the larva with 1 or 2 ml of water and dabbed it dry with a paper towel to avoid any confounding factors when weighing the larva. We then weighed the larvae individually using a micro-analytical balance (Mettler Toledo Model XPR6UD5, 0.5 µg readability) and transferred them into capped plates corresponding to either one of the environments described earlier (i.e. patchy or clumped). These plates were placed in an incubator set up at 25°C and 60% relative humidity. After 24 h, we weighed and recorded the final mass of larvae individually, following the same procedure described above. This experiment yielded data for 27 rovers and 31 sitters in the patchy environment, and 29 rovers and 22 sitters in the environment with clumped configuration of food.

### (e) Data analysis

We fitted competing models to investigate the effects of strain and environment on the proportion of area visited and the total distance travelled by the larvae in the test plates. Because the mass of the larvae might have affected their locomotion, including mass as a covariate in the models enabled us to correct for its potential confounding effect. Similarly, we modelled the effects of strain and environment on the growth of the larvae. The models that we fitted enabled us to test the main effects and the interactions between the independent variables. To do so, we fitted generalized least squares models (GLS) to account for heterogeneity in the data. This procedure was done using the function *glm* available in the ‘nlme’ package of the free software for statistical computing R v. 4.3.2 [23]. In addition, we used the function *lm* in R to fit ordinary least squares models, which did not account for heterogeneity in the data but enabled us to examine the same relationships tested with the GLS models. To evaluate the models’ goodness of fit, we used an information-theoretic approach such as AICc. We ranked the candidate models accordingly and selected the most likely one for inferences (lowest value of AICc).

To produce a complete report of our results and ensure that they are fully reproducible, we made all the analyses publicly available at https://dylan-padilla.github.io/foraging-experiments/.

## 3. Results

**Figure 1.**
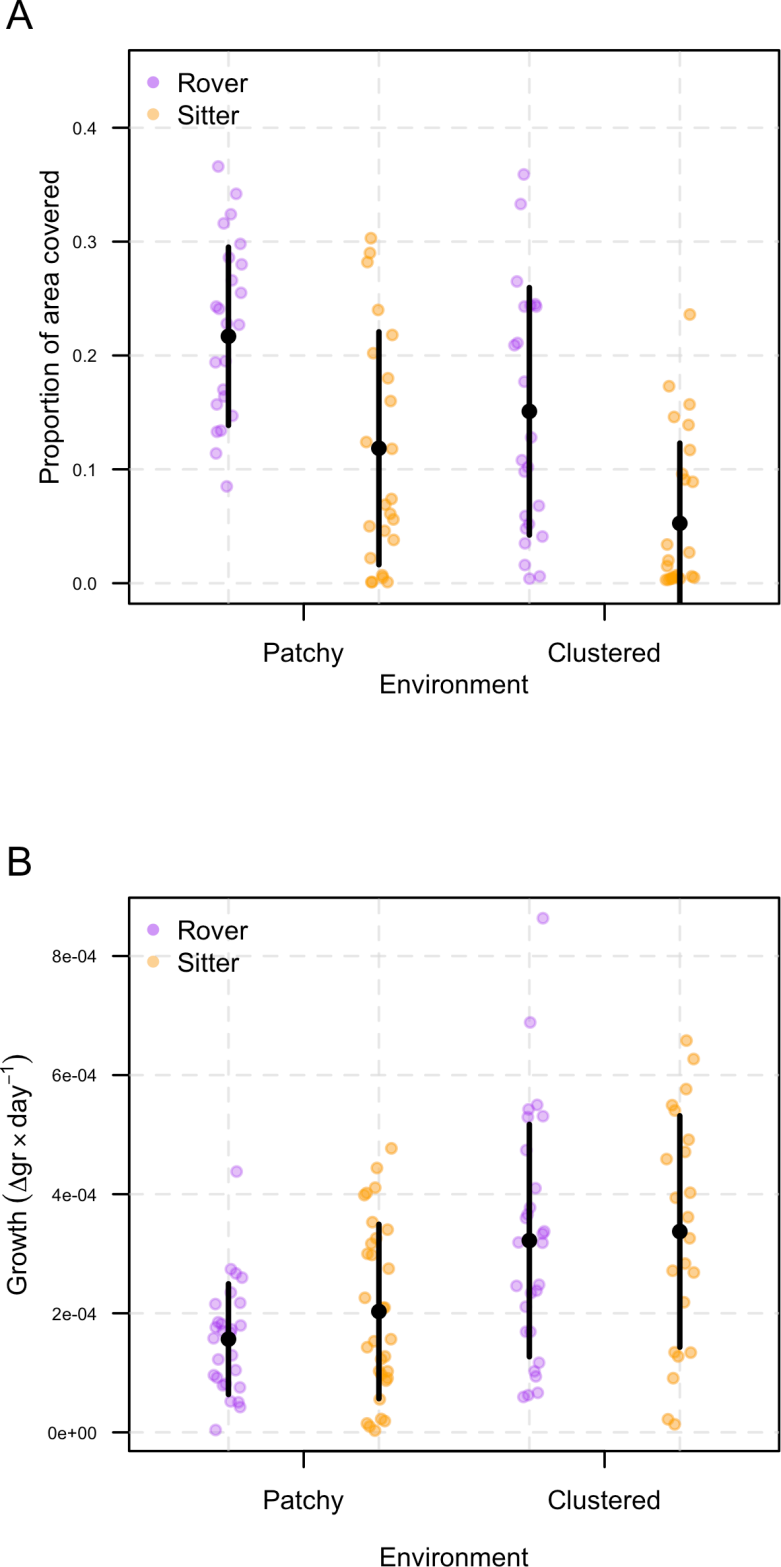
Comparisons among the predictors involved in the experimental design of the study. (A) Effects of strain and environment on the locomotor activity of the larvae. Within each environment, rovers covered a larger area than did sitters (p<0.001). Moreover, the area exploited by either strain was generally larger in the patchy environment (p=0.001). (B) Effects of strain and environment on larval growth. Larvae of both strains grew faster in the environment with a clumped configuration of food than they did in the patchy environment (p<0.001). Black dots represent the estimated means and the bars the standard deviation.

**Figure 2.**
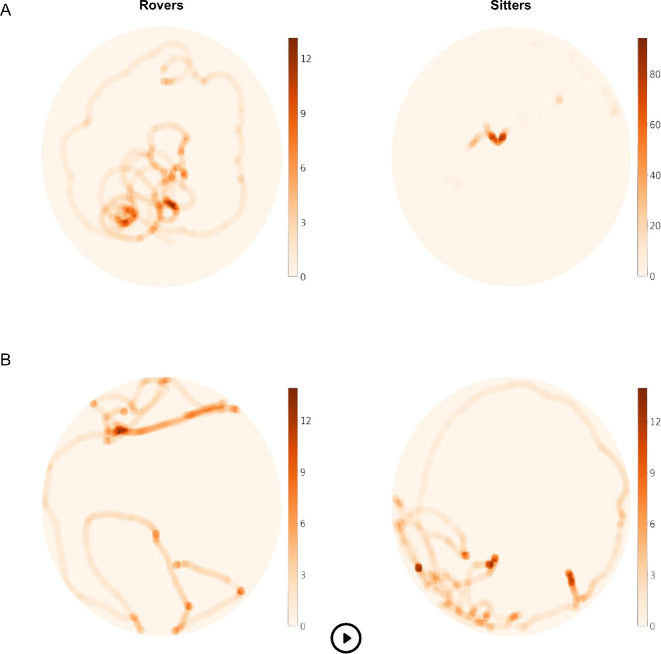
Animated visualization of the distance travelled by the larvae together with the proportion of time spent at specific sites of the test plates. A darker coloration indicates a longer proportion of time spent at a site (s). (A) Patchy environment. (B) Environment with clumped configuration of food. For an animated visualization in motion, please refer to https://dylan-padilla.github.io/foraging-experiments/.

Our analyses supported a model describing the effects of strain and environment on the proportion of area covered by the larvae while foraging (table 1). Two pieces of evidence aligned almost entirely with this result. First, within each environment, rovers covered a larger area than did sitters (β=−0.09;t=−5.17;p<0.001), yet the area exploited by either strain was generally larger in the patchy environment compared to that exploited in the environment with a clumped configuration of food (β=−0.07;t=3.47;p=0.001; figure 1A). Second, video tracking revealed that rovers generally travelled longer distances than did sitters (β=−576.22;t=−4.99;p<0.001), although we found no differences between environments (β=−192.69;t=−1.67;p=0.098; figure 2).

**Table 1.** Candidate models of the proportion of area covered by *Drosophila melanogaster* larvae as a function of strain and environment.

model	*K*	logLik	AICc	delta	weight
area visited ∼ strain + env	4.00	91.35	−174.24	0.00	0.57
area visited ∼ strain*env	5.00	91.69	−172.68	1.55	0.26
area visited ∼ mass*strain + env	6.00	91.98	−170.97	3.27	0.11
area visited ∼ mass*strain*env	9.00	94.84	−169.49	4.75	0.05

A star symbol (*) represents an interaction term.

Although rovers travelled farther and covered more area than sitters, both strains grew at similar rates. According to our analysis, a model describing only the effect of environmental patchiness on growth was strongly supported (table 2). Larvae of both strains grew faster in the environment with a clumped configuration of food than they did in the patchy environment ($\beta =1.57\times 10^{-4};t=-5.07;p<0.001$; figure 1B). In either type of environment, sitters grew slightly faster than rovers did, but the difference was not statistically significant. Interestingly, both rovers and sitters spent a significantly longer proportion of time at foraging sites when food was clumped (β=0.02;t=2.13;p=0.03; figure 2).

**Table 2.** Candidate models of *Drosophila melanogaster* larval growth as a function of strain and environment.

model	*K*	logLik	AICc	delta	weight
growth ∼ env, weight = vf3	6.00	786.64	−1560.45	0.00	1.00
growth ∼ strain, weight = vf3	6.00	777.09	−1541.36	19.10	0.00
growth ∼ strain+env, weight = vf3	7.00	778.05	−1540.99	19.46	0.00
growth ∼ strain*env, weight = vf3	8.00	769.43	−1521.42	39.03	0.00
growth ∼ strain*env, weight = vf2	6.00	766.77	−1520.71	39.74	0.00
growth ∼ strain*env	5.00	761.11	−1511.65	48.81	0.00
growth ∼ strain*env, weight = vf1	6.00	761.27	−1509.73	50.73	0.00

A star symbol (*) in the models represents an interaction term. The ’weight’ argument describes the within-group heteroscedasticity structure as follows: vf1 = varIdent(∼ 1|strain), vf2 = varIdent(∼ 1|env), vf3 = varIdent(∼ 1|strain * env).

## 4. Discussion

Our results suggest that foraging behaviour is a plastic trait shaped by the distribution of food in the environment, which aligns with the findings of recent investigations (e.g. [18,25]). Based on theoretical models, we predicted that both strains would cover more area in a patchy environment compared to a clumped environment. This expectation relies on the fact that food was spread throughout a larger area in a patchy environment; therefore, the energy gained by moving would likely surpass that of remaining in a patch. By contrast, the clumped environment contained a single clump of food in one location. In that case, leaving the sole clump of food would reduce the rate of energy gain to zero. Following the same notion, we expected a patchy environment to cause a more pronounced difference in locomotion between strains. This predicted interaction between strain and environment was not supported by our results. These findings suggest that the relationship between the configuration of resources in the environment, the density of resources and the locomotion of organisms is likely more complex than expected.

A key assumption underlying foraging theory is that organisms behave to maximize the net energy intake during foraging. Because the rate of energetic gain or loss greatly affects fitness [10–13], assessing which foraging strategy yields the highest surplus energy in a given environment becomes crucial to understanding the evolution of behaviour [6,26]. Often, investigating the connection between the phenotype being optimized (i.e. foraging behaviour) and a ‘fitness component’—such as growth—offers the opportunity to accomplish such a task [27]. When testing the effects of foraging behaviour on growth, we discovered that an environment with clumped configuration of food stimulated a faster growth in both strains than did a patchy environment. Interestingly, however, both strains grew to similar sizes in either environment even though rovers generally covered larger areas than did sitters while foraging. These observations were not entirely consistent with our predictions, because we expected the rover strain to decrease its locomotor activity and grow more slowly in the environment with clumped configuration of food. However, our findings are consistent with previous observations supporting the absence of a relationship between foraging behaviour and life-history traits [15,16], adding more evidence against the general belief that foraging polymorphism leads to variation in life-history traits [27–29]. Because food was relatively abundant in each environment, and strains were tested individually, the density of food was not depleted at the same rate as it would be in the presence of competitors. Thus, there may have been little incentive to minimize daily foraging time and energy by rovers. The minimization of daily energy expenditure might not be necessary when food is relatively abundant, but a highly efficient energy-expensive foraging strategy, such as searching for food, would become advantageous as it saves time for other critical activities [30].

Based on the results of this study, the question remains regarding which foraging strategy is better suited to maximize energy intake in a particular environment. Previous research indicates that actively searching for food could be advantageous when food is either patchy or clumped. Two kinds of mathematical models provide support for this claim. As described above, an energetically demanding but efficient strategy of searching for food enables animals to save time for other activities, such as reproduction [30]. In addition, eco-evolutionary models suggest that the ability of rovers to travel longer distances while foraging influences their dispersal (‘high-dispersing’ strategy). In its simplest form, dispersal can be defined as any movement with potential for genetic mixing [31], facilitating local adaptation via founder events, gene flow, and life-history trade-offs [32]. If actively pursuing food promotes higher dispersal, such behaviour may facilitate the ability of organisms to adapt and colonize new environments [33]. Although the dispersal ability of a larva may be limited, previous investigations suggest that behaviours that are expressed early in life are closely correlated with a set of life-history traits that enhance the ability to colonize new environments and often retain flexibility in expression throughout an organism’s life (e.g. [34]). In insects, for instance, some species exhibit populations with individuals that express winged dispersive and wingless non-dispersive morphs [35–37]. In other animals, such as birds, individuals with a great ability to disperse also have behaviours that enhance their survival and competitive ability in novel environments [38,39].

Taken together, our study implemented a simple yet robust way to test some of the long-standing predictions of the relationship between foraging behaviour and life history. We suggest that foraging behaviour is a highly plastic trait moulded by the configuration of food in the environment. Our findings support the notion that animals enhance their energy intake by adjusting their locomotion according to environmental heterogeneity. In wild populations of *D. melanogaster*, this could mean that rovers can influence the rate of population spread to a higher extent than would sitters. As such, foraging actively could have important implications for colonization or range expansion to novel habitats through the subsequent evolution of life-history traits. Although the interpretation of our results is limited to the larvae of *D. melanogaster*, evidence of correlated evolution of foraging behaviour in larvae and adults enables us to make predictions at both developmental stages [9,20]. Importantly, fitness should be measured over the entire life of an organism. Thus, the possibility exists that the appropriate time interval to notice important differences in growth is over a longer period than the one considered in our study.

## Data Availability

A fully reproducible workflow of the data analyses, including R scripts and additional supporting material, is available from the following repositories: Github [40]. Dryad Digital Repository [41]. Supplementary material is available online [42].
